# Intravitreal aflibercept alone versus combination with dexamethasone phosphate for diabetic macular edema: A randomized phase-2 clinical trial

**DOI:** 10.5935/0004-2749.2025-0098

**Published:** 2025-06-24

**Authors:** Valesca Castro Neri, Rodrigo Pessoa Cavalcanti Lira, Andrea Andrade Azevedo de Vasconcelos, Pedro Henrique Lasalvia Jorge, Gabriel Rocha Lira, Victor da Cunha Lima Almeida

**Affiliations:** 1 Universidade Federal de Pernambuco, Recife, PE, Brazil

**Keywords:** Diabetic macular edema, Aflibercept, Dexamethasone sodium phosphate, Intravitreal injection, Visual acuity, Central macular thickness, Intraocular pressure

## Abstract

**Purpose:**

To compare the short-term (3-month) outcomes of intravitreal aflibercept injections versus intravitreal aflibercept combined with dexamethasone sodium phosphate in treating diabetic macular edema.

**Methods:**

In this Phase-2 clinical trial, 16 eyes of 16 participants with diabetic macular edema were randomly assigned to one of 2 groups. Participants in the aflibercept monotherapy group received 2 mg of intravitreal aflibercept (0.05 mL), while those in the combination therapy group received 2 mg of intravitreal aflibercept (0.05 mL) plus 0.04 mg dexamethasone sodium phosphate (0.01 mL). Identical injections were repeated after 30 and 60 days. The primary outcome was the change in central macular thickness, as measured by optical coherence tomography, from baseline to 1 month after the last injection. Secondary outcomes included changes in best-corrected visual acuity and intraocular pressure over the same period.

**Results:**

The mean baseline central macular thickness was 444 ± 86 µm in the combination therapy group and 394 ± 96 µm in the aflibercept monotherapy group (p=0.293). By day 90, the mean reduction in central macular thickness was significantly greater in the combination therapy group (176 ± 129 µm) compared to the aflibercept monotherapy group (54 ± 49 µm; p=0.034). Best-corrected visual acuity also improved significantly more in the combination therapy group, with a median gain of 0.31 ± 0.16 LogMAR, whereas the aflibercept monotherapy group experienced a minimal change (-0.06 ± 0.13 LogMAR; p=0.020). Intraocular pressure remained stable in both groups, with no significant difference (p=0.855). None of the participants developed elevated intraocular pressure (>21 mmHg) or required ocular hypotensive medications. No significant ocular or systemic adverse events were reported.

**Conclusion:**

The addition of dexamethasone sodium phosphate to the standard intravitreal aflibercept regimen for diabetic macular edema can improve short-term structural and functional outcomes.

**Trial registration:**

Brazilian Clinical Trials Registry (RBR-7468j4q)

## INTRODUCTION

Diabetic macular edema (DME) results from the accu-mulation of subretinal and intraretinal fluid, caused by the breakdown of the blood-retinal barrier and the leakage of fluid from abnormal perifoveal capillaries and microaneurysms, due to microvascular changes induced by diabetes^(1)^. Several inflammatory factors have been implicated in the pathogenesis of DME, including vascular endothelial growth factor (VEGF), interleukin-6 (IL-6), interleukin-8 (IL-8), and monocyte chemotactic protein-1 (MCP-1). These inflammatory mediators, associated with elevated VGEF levels, are responsible for the onset and aggravation of macular edema^(2)^. Evidence suggests that the late stage of DME may be more driven by inflammatory activity than by angiogenic action^(3^-^5)^. According to the World Health Organization (WHO), approximately 537 million people had type 2 diabetes in 2021, and this number is projected to increase to 643 million by 2030. Diabetic retinopathy complications account for approximately 2% of all cases of visual impairment and 2.6% of cases of blindness. DME is considered the leading cause of visual loss in diabetic patients, affecting approximately 6.8% of patients^(6)^.

Several studies have compared combination treatments of anti-VEGF agents (aflibercept, bevacizumab, or ranibizumab) with corticosteroids (triamcinolone or biode-gradable dexamethasone implant, Ozurdex^®^) versus anti-VEGF monotherapy for DME. The results showed that combination therapy decreased central macular thickness (CMT) and reduced the need for injection^(7^-^11)^. Combination therapy also provided longer intervals between injections and faster recovery of best-corrected visual acuity (BCVA)^(10)^, particularly in patients with prominent inflammation^(9)^, pseudophakia^(9)^, or those refractory to anti-VEGF monotherapy^(8^,^10)^. However, this combination therapy led to increased intraocular pressure (IOP) in up to 30% of patients, and was associated with a greater progression of cataract in phakic eyes and high costs, particularly of the biodegradable dexamethasone implant^(11^-^13)^.

The dexamethasone sodium phosphate (DSP) solution offers a very affordable option (US$ 2) for treating DME. Fonseca et al.^(14)^ and Lira et al.^(15)^ investigated the efficacy of DSP in DME. Results showed improvements in BCVA in some patients and a significant reduction in CMT within 7 days. However, the therapeutic effect waned over the first month. Notably, intravitreal DSP solution did not significantly alter IOP, and no severe ocular or systemic adverse events were observed. These findings suggest that DSP may be an affordable therapeutic option for short-term management of DME^(14)^.

Karimi et al. evaluated the short-term effects of combining intravitreal DSP with bevacizumab (IVB) in patients with anti-VEGF-refractory DME. In this study, patients were divided into two groups: one receiving IVB + DSP and the other receiving IVB alone. The results showed that combination therapy (IVB + DSP) yielded significantly greater reductions in CMT and improvements in BCVA compared to IVB alone. Notably, no significant difference in IOP was observed between the two groups^(16)^.

The objective of the present study was to compare the 3-month outcomes of intravitreal aflibercept monotherapy versus aflibercept plus DSP combination therapy in patients with DME.

## METHODS

### Study design

This Phase 2 clinical trial was approved by the Federal University of Pernambuco (CAAE 66066122.8.0000.5208) and registered with the Brazilian Clinical Trials Registry (RBR-7468j4q). The study was conducted at a private clinic in Recife, Brazil, where all participants provided written informed consent.

### Participants

A total of 16 participants were divided into 2 groups of 8 individuals each. One group received aflibercept (IVA) [Eylea^®^, Bayer™, USA] monotherapy, while the other group received IVA plus DSP combination therapy. Block randomization of 4 participants (2 from each group) was adopted. If a participant had DME in both eyes, only one eye was included in the study, selected by random sampling. The study employed a masked design, where both participants and data collectors were blinded to the group identity.

### Inclusion and exclusion criteria

The inclusion criteria were as follows: (1) patients aged ≥18 years with clinically significant DME in one or both eyes according to ETDRS guidelines; (2) BCVA between 1.3 and 0.3 LogMAR; (3) CMT ≥ 300 µm, measured by spectral-domain optical coherence tomography (SD-OCT) (Heidelberg™, Germany).

The exclusion criteria were: (1) history of any treat-ment for DME in the preceding 4 months; (2) panretinal photocoagulation (PRP) or any ocular surgery performed in the preceding 4 months; (4) history of pars plana vitrectomy; (5) history of open-angle glaucoma or corticosteroid-induced elevated IOP requiring antiglaucoma or antihypertensive ocular treatment; (6) IOP >21 mmHg; (7) history of allergy to any product used in the procedure; (8) presence of tractional retinal detachment.

### Intervention

The treatment protocol consisted of intravitreal injections administered on days 0, 30, and 60. One group received 0.05 mL (2 mg) of IVA, while the other group received a combination of 0.05 mL (2 mg) of IVA and 0.01 mL (0.04 mg) of DSP. Injections were administered using a 0.5 mL insulin syringe with a 0.3 mm × 8 mm needle (BD SafetyGlide™ Insulin, BD™, USA) (a low dead space syringe with a fixed needle), under microscopic guidance. Topical anesthesia was achieved with two drops of proxymetacaine eye drops. Before the procedure, skin antisepsis was performed with topical chlorhexidine, followed by conjunctival antisepsis with 5% topical povidone, and the use of a disposable sterile surgical drape and sterile blepharostat. Post-procedure, two drops of moxifloxacin eye drops were instilled, and lubricating eye drops (carboxymethylcellulose) were prescribed for ongoing use.

### Outcomes

A comprehensive ophthalmological evaluation was performed during the pre-intervention screening visit, including BCVA, anterior segment biomicroscopy, applanation tonometry, fundus examination with a 78 diopter lens, and SD-OCT. CMT was assessed using SD-OCT with seven horizontal lines (30° × 5° area) centered on the fovea, comprising 1536 scans per line at 240 µm intervals. Follow-up visits were scheduled at 3, 7, 30, 60, and 90 days, with repeated assessments of BCVA, anterior segment biomicroscopy, applanation tonometry, fundus examination with a 78 diopter lens, and SD-OCT. Glycated hemoglobin measurements were obtained at screening. The primary outcome measure was the change in CMT, as measured by SD-OCT, from baseline to one month after the last injection. Secondary outcome measures included changes in BCVA and IOP from baseline to one month after the last injection.

### Sample size calculation & statistical analysis

A sample size of 7 individuals per group was calculated assuming a power greater than 90% and a probability of type 1 error less than 0.05% (two-tailed) to detect a difference of 120 µm in the CMT (with a standard deviation of 60 µm, and allocation radius of 1:1) between the groups. However, to account for attrition during follow-up, 16 subjects were enrolled.

Data were summarized using descriptive statistics. The normality of distribution of continuous variables was assessed using the Shapiro-Wilk test. Normally distributed continuous variables were presented as mean ± standard deviation (SD), while non-normally distributed continuous variables were presented as median (interquartile range (IQR). Between-group differences regarding non-normally distributed variables were assessed for statistical significance using the Wilcoxon test. Categorical variables were compared using the chi-square test or the Fisher-Freeman-Halton exact test. Statistical analyses were conducted using SPSS version 21 (IBM Corporation, Armonk, NY, USA) and Epi Info version 5.5.8 (CDC, USA). Two-tailed p-values <0.05 were considered indicative of statistical significance.

## RESULTS

All participants completed all the scheduled visits on days 0, 3, 7, 30, 60, and 90 following their first intravitreal injection. The qualitative and quantitative pre-injection characteristics of each group are described in Tables 1 and 2, respectively.

**Table 1:** Qualitative characteristics of each group before intervention

Characteristics	Aflibercept + DSP group	Aflibercept group	p-value^a^
	(n=8)	(n=8)	
Right eye	4/8	5/8	0.500
Sex	5/8	6/8	0.500
Dyslipidemia	5/8	5/8	0.696
Arterial hypertension	8/8	7/8	0.500
Coronary artery disease	2/8	2/8	0.715
History of intravitreal Anti-VEGF	7/8	6/8	0.500
History of intravitreal steroid	0/8	0/8	-
History of retinal photocoagulation	2/8	1/8	0.500
Cataract surgery	2/8	2/8	0.715
Disorganization of the inner retinal layers	8/8	7/8	0.500
Elipsoid zone discontinuity	7/8	5/8	0.285
Foveal exudates	7/8	8/8	0.500
Subfoveal exudates	2/8	3/8	0.500
Hiperreflective foci	8/8	8/8	-
Subfoveal fluid	2/8	2/8	0.715
Extrafoveal epiretinal membrane	2/8	1/8	0.500
Foveal intraretinal cysts	7/8	6/8	0.500
Extrafoveal intraretinal cysts	7/8	7/8	0.767
Subfoveal EPR atrophy	3/8	3/8	0.696

a Fisher’s Exact Test.

DSP= dexamethasone sodium phosphate.

**Table 2:** Quantitative characteristics of each group before intervention

Characteristics	Aflibercept + DSP group		Aflibercept group		p-value
	Mean	SD	Mean	SD	
Time elapsed since diagnosis of diabetes mellitus (years)	13	5.8	13.8	9.8	0.855^a^
Glycated hemoglobin (%)	7.41	1.3	8.16	0.7	0.188^a^
Macular thickness on SD-OCT (µm)	444	86	394	96	0.293^a^
	**Median**	**IQR**	**Median**	**IQR**	
Age (years)	62	5	63.5	8	0.792^b^
Corrected distance visual acuity (CDVA) (LogMAR)	0.64	0.46	0.48	0.07	0.234^b^
Intraocular Pressure (mmHg)	17	2	16	3	0.591^b^

a Student’s *t*-test for Independent Samples.

b Mann-Whitney test.

SD-OCT= spectral domain optical coherence tomography; SD= standard deviation; IQR= interquartile range; DSP= dexamethasone sodium phosphate.

**Figure 1:**
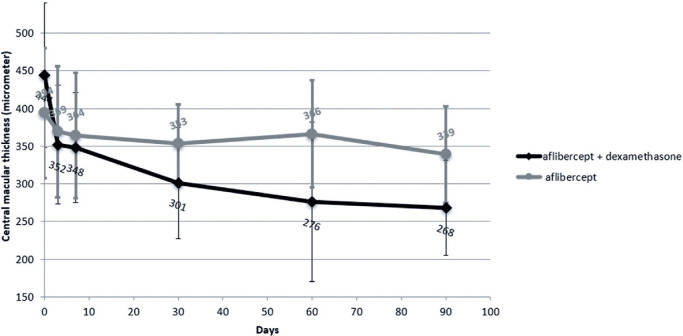
Changes in mean central macular thickness over 90 days in patients treated with intravitreal aflibercept monotherapy versus intravitreal aflibercept plus dexamethasone sodium phosphate

The mean difference in CMT on SD-OCT from baseline to day 90 post-injection in the IVA + DSP group and the IVA group was 176 ± 129 µm and 54 ± 49 µm, respectively (p=0.034) (Figure 1 and Table 3).

**Table 3:** Changes in central macular thickness in the two groups between various time points

Time points of SD-OCT evaluation	Aflibercept + DSP group			Aflibercept group			p^^1^^	p^^2^^
	Mean (%)	Mean (µm)	SD (µm)	Mean (%)	Mean (µm)	SD (µm)		
Day 0 and 3	(29)	92	63	(6)	25	37	0.043^a^	0.021^a^
Day 0 and 7	(30)	96	30	(8)	30	46	0.013^b^	0.029^a^
Day 0 and 30	(51)	143	93	(12)	41	49	0.013^b^	0.016^a^
Day 0 and 60	(73)	167	126	(9)	29	43	0.033^a^	0.017^a^
Day 0 and 90	(79)	176	129	(15)	54	49	0.045^a^	0.034^a^

a Student’s t-test for independent samples;

b Mann-Whitney test.

SD-OCT= spectral domain optical coherence tomography; DSP= dexamethasone sodium phosphate; SD= standard deviation.

p1= Statistical significance for proportions.

p2= Statistical significance for absolute values.

**Figure 2:**
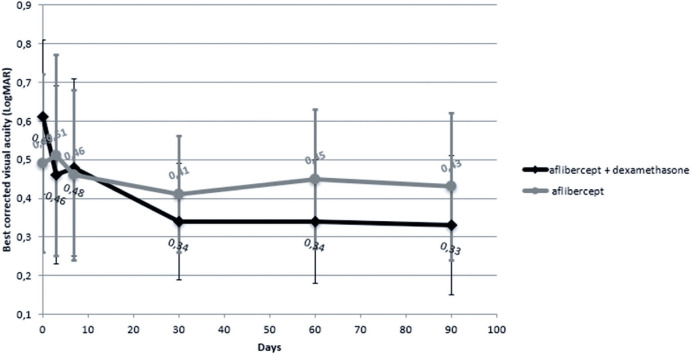
Changes in mean best corrected visual acuity over 90 days in patients treated with intravitreal aflibercept monotherapy versus intravitreal aflibercept plus dexamethasone sodium phosphate

The median (IQR) change in BCVA from baseline to day 90 post-injection was 0.31 (0.16) LogMAR in the IVA + DSP group and -0.06 (0.13) LogMAR in the IVA group (p=0.020) (Figure 2 and Table 4).

**Table 4:** Changes in best corrected visual acuity in the two groups between various time points

Time points of BCVA evaluation	Aflibercept + DSP group		Aflibercept group		p-value
	Median (LogMAR)	IQR (LogMAR)	Median (LogMAR)	IQR (LogMAR)	
Day 0 and 3	0.00	0.14	0.00	0.21	0.032^a^
Day 0 and 7	0.00	0.14	0.01	0.14	0.105^a^
Day 0 and 30	0.24	0.23	0.00	0.05	0.032^b^
Day 0 and 60	0.30	0.10	-0.06	0.13	0.013^a^
Day 0 and 90	0.31	0.16	-0.06	0.13	0.020^a^

a Mann-Whitney Test;

b Student’s *t*-test for independent samples;

BCVA= best corrected visual acuity; IQR= interquartile range; DSP= dexamethasone sodium phosphate.

**Figure 3:**
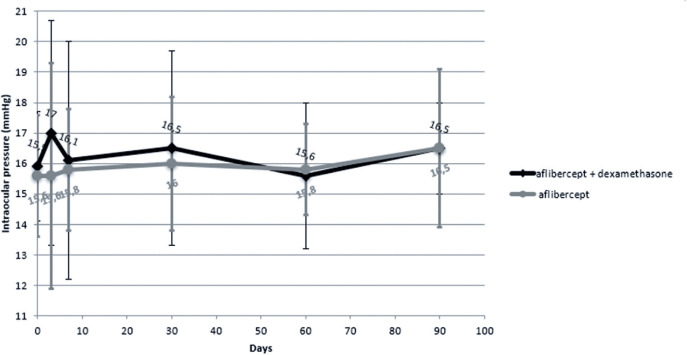
Changes in mean intraocular pressure over 90 days in patients treated with intravitreal aflibercept monotherapy versus intravitreal aflibercept plus dexamethasone sodium phosphate

The mean change in IOP from baseline to day 90 post-injection was -0.6 ± 2 mmHg in the IVA + DSP group and -0.9 ± 3.2 mmHg in the IVA group (p=0.855) (Figure 3 and Table 5).

**Table 5:** Changes in intraocular pressure in the two groups between various time points

Time points of IOP evaluation	Aflibercept + DSP group		Aflibercept group		p-value
	Mean (mmHg)	SD (mmHg)	Mean (mmHg)	SD (mmHg)	
Day 0 and 3	-1.1	4.2	0.0	2.7	0.532^a^
Day 0 and 7	-0.3	3.0	-0.1	2.8	0.932^a^
Day 0 and 30	-0.6	3.1	-0.4	2.6	0.863^a^
Day 0 and 60	0.3	2.1	-0.1	2.0	0.719^a^
Day 0 and 90	-0.6	2.0	-0.9	3.2	0.855^a^

a Student’s *t*-test for independent samples.

IOP= intraocular pressure; DSP= dexamethasone sodium phosphate; SD= standard deviation.

There was no significant change in IOP in either group. None of the eyes in either group showed an IOP increase of >5 mmHg. No cases of endophthalmitis, uveitis, vitreous hemorrhage, retinal rupture, cataract development, or retinal detachment were observed in either group. No systemic adverse effects were reported.

## DISCUSSION

This study represents the first published clinical trial evaluating the intravitreal use of a combination of aflibercept and DSP solution for treating DME. The IVA + DSP group demonstrated greater improvements in BCVA and a more significant reduction in CMT over the 90 days following the first intravitreal injection, compared to the IVA-alone group.

The mean reduction in CMT on day 90 was greater in the IVA + DSP group compared to the IVA-alone group. This finding aligns with Lin et al.’s analysis, which evaluated aflibercept combined with a slow-release 0.7 mg dexamethasone intravitreal implant. They reported a significant CMT reduction from baseline in the combination group, an effect not observed in the control group^(8)^. In the present trial, the CMT reduction in the IVA + DSP group was most pronounced at day 30, with an average reduction of 143 ± 93 µm from baseline, and continued to decrease, reaching a 176 ± 129 µm mean reduction at day 90. Similarly, Lin et al.^(8)^ noted the most significant CMT reduction in the combination group during the first month of their clinical trial, a pattern supported by Ozsaygılı et al.^(9)^, Barakat et al.^(7)^, and Limon et al.^(10)^. Ozsaygılı et al. reported significant CMT reductions in both groups, but after the first month, the combination group exhibited a significantly greater decrease than the aflibercept-alone group, an effect that persisted up to 9 months (p<0.001)^(9)^. Similarly, Barakat et al. observed a rapid CMT reduction at 4 weeks in the group receiving triamcinolone acetonide suspension combined with aflibercept, with a statistically significant difference compared to the aflibercept-alone group (p=0.047), despite both groups showing a decrease in mean CMT in the subsequent 16 weeks^(7)^. At 24 weeks, CMT stabilized in both groups, with the combination group achieving a greater final CMT reduction (226.5 µm versus 176.1 µm, p=0.035)^(7)^. In Limon et al.’s analysis, the combination group exhibited an average CMT reduction of 142.25 µm from baseline to month 3 (p<0.05), with the baseline thickness of 432.42 ± 113.6 µm decreasing to 279.22 ± 106.32 µm within the first month. In contrast, the control group showed an average CMT reduction of 17.30 µm from baseline to month 3 (p>0.05)^(10)^.

Our findings on CMT reduction align with those of Karimi et al., whose interventional case series reported a greater mean CMT reduction in the combination group compared to the bevacizumab alone group (109.88 ± 156.25 µm vs 43 ± 113.7 µm, p=0.03)^(16)^.

The improvement in BCVA in the IVA + DSP group was both clinically and statistically significant, with a gain of 3 lines on the ETDRS chart between baseline and day 90 post-injection, surpassing the outcomes in the IVA-alone group. These findings compare favorably to those reported by Lin et al., who investigated the combination of aflibercept with a slow-release 0.7 mg dexamethasone intravitreal implant^(8)^. In their study, 50 eyes received monthly aflibercept intravitreal injections, while 52 eyes received monthly aflibercept injections combined with dexamethasone implant. Although both groups demonstrated improved BVCA over 6 months, the gains were not significantly different between groups. Notably, the combination group exhibited greater monthly BCVA improvements between 30 and 60 days, but this advantage was lost by 90 days, with equivalent outcomes between groups^(8)^.

Ozsaygılı et al. evaluated the one-year outcomes of an interventional trial involving 82 eyes of 82 patients with treatment-naive DME^(9)^. The study divided patients into 2 groups: 43 eyes received 2 mg intravitreal aflibercept monotherapy for 3 months, followed by a pro re nata (PRN) protocol, while 39 eyes received PRN aflibercept injections combined with a slow-release dexamethasone intravitreal implant at the initiation of treatment. Both groups demonstrated significant improvements in BCVA compared to baseline. Notably, the combination group exhibited significantly higher BCVA in the first few months, consistent with our findings. However, after six months, BCVA values converged, with similar outcomes between groups. At the end of 12 months, the combination group achieved an average gain of 11.6 ETDRS letters, compared to 9.3 letters in the aflibercept-alone group (p=0.240)^(9)^.

Limon’s non-randomized prospective study evaluated the efficacy of combining a slow-release dexamethasone intravitreal implant with bevacizumab versus bevacizumab monotherapy in patients with persistent DME^(10)^. The combination group demonstrated significant improvements in BCVA, with an increase from a mean of 44 ± 19 letters (ETDRS) at baseline to 59 ± 18 letters at one month (p<0.05), 57 ± 19 letters at 2 months (p<0.05), and 55 ± 18 letters at three months (p<0.05). The control group showed no significant improvement in BCVA (43 ± 19 letters at baseline and 44 ± 18 letters at 3 months)^(10)^. Our findings align with Limon’s results, particularly in demonstrating greater visual gains in the combination group during the initial weeks of the study^(10)^.

A 2023 study by Karimi et al. investigated the efficacy of intravitreal DSP in combination with bevacizumab for treating refractory DME. Their prospective interventional case series included 81 eyes of 81 individuals, divided into two groups. The combination group (40 eyes) received 1.25 mg intravitreal bevacizumab plus 8 mg/2 mL intravitreal DSP, while the control group (41 eyes) received bevacizumab alone^(16)^. Evaluations were conducted at baseline and one month after the third injection. Consistent with our findings, the combination group showed significant improvement in BCVA, with a mean change of 0.13 ± 0.23 logMAR (p=0.001). In contrast, the bevacizumab alone group showed no statistically significant improvement, with a mean change of 0.01 ± 0.17 logMAR (p=0.805). The between-group difference in this respect was statistically significant (p=0.008)^(16)^.

Our study found no significant between-group diffe-rence in terms of IOP change between baseline and day 90 post-injection. Similarly, Karimi et al.’s study reported no significant IOP change between groups. On average, IOP decreased by 0.65 ± 2.65 mmHg in the combination therapy group and increased by 0.46 ± 2.05 in the bevacizumab alone group (p=0.028)^(16)^.

Some limitations of our study should be acknowledged. The small sample size may have compromised the reliability and generalizability of our findings. Additionally, the short follow-up duration prevented the assessment of the long-term maintenance of BCVA and CMT reduction. Furthermore, it hindered our ability to observe cataract development in the medium and long term. Moreover, as participants were only examined up to 30 days after the last intravitreal injection, we could not determine if there was a difference in the need for retreatment between groups.

In conclusion, the study’s findings suggest that adding dexamethasone sodium phosphate to the intravitreal aflibercept regimen improves visual and structural outcomes in individuals with DME within the first 3 months, without increasing the risk of endophthalmitis, elevated IOP, cataract, or intraocular inflammation. Longer-term studies with a larger sample size are necessary to confirm these findings.

## References

[1] Sohn HJ, Han DH, Kim IT, Oh IK, Kim KH, Lee DY (2011). Changes in aqueous concentrations of various cytokines after
intravitreal triamcinolone versus bevacizumab for diabetic macular
edema. Am J Ophthalmol.

[2] Whitcup SM, Cidlowski JA, Csaky KG, Ambati J. (2018). Pharmacology of corticosteroids for diabetic macular
edema. Invest Ophthalmol Vis Sci.

[3] Daruich A, Matet A, Moulin A, Kowalczuk L, Nicolas M, Sellam A (2018). Mechanisms of macular edema: Beyond the surface. Prog Retin Eye Res.

[4] Urias EA, Urias GA, Monickaraj F, McGuire P, Das A. (2017). Novel therapeutic targets in diabetic macular edema: Beyond
VEGF. Vision Res.

[5] Zhang X, Wang N, Schachat AP, Bao S, Gillies MC. (2014). Glucocorticoids: structure, signaling and molecular mechanisms in
the treatment of diabetic retinopathy and diabetic macular
edema. Curr Mol Med.

[6] Gabrielle PH, Mehta H, Barthelmes D, Daien V, Nguyen V, Gillies MC (2023). From randomised controlled trials to real-world data: Clinical
evidence to guide management of diabetic macular oedema. Prog Retin Eye Res.

[7] Barakat MR, Wykoff CC, Gonzalez V, Hu A, Marcus D, Zavaleta E (2021). Suprachoroidal CLS-TA plus Intravitreal Aflibercept for Diabetic
Macular Edema: A Randomized, Double-Masked, Parallel-Design, Controlled
Study. Ophthalmol Retina.

[8] Lin TC, Chung YC, Hsu TK, Huang HW, Huang YM, Chou YC (2022). Therapeutic effect of simultaneous intravitreal dexamethasone and
aflibercept on diabetic macular edema. Acta Diabetol.

[9] Ozsaygılı C, Bayram N. (2024). Does dexamethasone implant combination with aflibercept
monotherapy affect one-year outcomes in treatment-naive diabetic macular
edema with inflammatory biomarkers?. Int Ophthalmol.

[10] Limon U. (2021). Early effect of simultaneous intravitreal dexamethasone and
bevacizumab combination treatment in patients with persistent diabetic
macular edema. J Fr Ophtalmol.

[11] Maturi RK, Glassman AR, Liu D, Beck RW, Bhavsar AR, Bressler NM (2018). Effect of adding dexamethasone to continued ranibizumab treatment
in patients with persistent diabetic macular edema. JAMA Ophthalmol.

[12] Chen C, Wang Z, Yan W, Lan Y, Yan X, Li T (2023). Anti-VEGF combined with ocular corticosteroids therapy versus
anti-VEGF monotherapy for diabetic macular edema focusing on drugs injection
times and confounding factors of pseudophakic eyes: A systematic review and
meta-analysis. Pharmacol Res.

[13] Cheng Z, Liu X. (2024). Comparing the efficacy of glucocorticoids and anti-VEGF in
treating diabetic macular edema: systematic review and comprehensive
analysis. Front Endocrinol.

[14] ALA Panetta H, Nascimento MA, Lira RP, Arieta CE (2019). Effect of intravitreal dexamethasone solution on the reduction of
macular thickness in pseudophakic diabetic patients in a public hospital in
Brazil: A randomized clinical trial. Clin Ophthalmol Auckl NZ.

[15] Lira Rodrigo PC, Oliveira Armando YS, Lira Gabriel R (2025). Cost-effective treatment for diabetic macular edema using
dexamethasone sodium phosphate. Arq Bras Oftalmol.

[16] Karimi S, Karrabi N, Hassanpour K, Amirabadi A, Daneshvar K, Nouri H (2023). The additive effect of intravitreal dexamethasone combined with
bevacizumab in refractory diabetic macular edema. J Fr Ophtalmol.

